# Effects of different phosphorus‐free water‐retaining agents on the quality of frozen tilapia fillets

**DOI:** 10.1002/fsn3.2686

**Published:** 2021-12-28

**Authors:** Min Li, Jing Luo, Ying Zhang, Ke Zhang, Zhi Qiang Guan, Chang Ming Ling

**Affiliations:** ^1^ College of Mechanical and Power Engineering Guangdong Ocean University Zhanjiang China; ^2^ College of Food Science and Technology Guangdong Ocean University Zhanjiang China; ^3^ Guangdong Provincial Key Laboratory of Aquatic Product Processing and Safety Guangdong Ocean University Zhanjiang China

**Keywords:** food quality, frozen storage, phosphate‐free water‐retaining agent, Tilapia fillet

## Abstract

Tilapia is an economically important fish worldwide, but its quality is affected by storage practices. To improve the quality of tilapia fillets during frozen storage, we examined the effect of pretreatment with various combinations of different concentrations of trehalose, potassium bicarbonate, and chitosan. Following pretreatment, we analyzed the tilapia fillets using quality indicators, including soaking weight gain, coating weight gain, water‐holding capacity, thawing loss, pH, Ca^2+^‐ATPase activity, and texture characteristics. Water distribution was analyzed using low‐field nuclear magnetic resonance and the optimal combination of water‐retaining agents was obtained using an L_8_(2^7^) orthogonal experiment. The results showed that trehalose, potassium bicarbonate, and chitosan improved fillet quality at pretreatment concentrations of 5%–8%, 1.0%, and 0.5%, respectively. The optimal combination was 4% trehalose plus 1.2% potassium bicarbonate plus 0.2% chitosan. The Ca^2+^‐ATPase activity and mastication property of the frozen fillets that were pretreated with the optimized formulation were 1.39 μmol Pi/mg protein·h and 8.55 mJ, respectively, which were 43.3% and 80.0% greater, respectively, than that of the control group. Using a suitable concentration and combination of water‐retaining agents cannot only lock‐in the internal water content of frozen tilapia fillets but also improve their quality during frozen storage. These results can inform practical storage practices of similar aquatic products.

## 1 INTRODUCTION

Tilapia is an economically important fish widely cultivated worldwide. Because of its rich nutrition (Zhao et al., 2015), fresh tilapia is especially prone to spoilage by microorganisms and enzymatic activity. To improve the quality of tilapia fillets and prolong their storage period, they should be stored at low temperature. Currently, the low‐temperature storage methods used for tilapia fillets mainly include frozen storage (Cheng et al., 2017; Chu & Xie, 2020), cold storage (Chang et al., 2019), and microfreezing (Liu et al., 2013). Slice freezing is one of the main forms of tilapia processing (Zhao et al., 2017); however, frozen fish fillets are prone to water volatilization, a decrease in quality and water‐holding rate, protein dehydration, and denaturation during storage, transportation, circulation, and sale (Chen et al., 2011).

Compared with other storage methods, frozen storage has the advantages of low costs and long storage times, which readily allow for adjustments to market demand in the off‐seasons and peak periods. Therefore, researchers have focused on developing methods to retain quality and limit variation in tilapia fillets during frozen storage. These studies found that the appropriate pretreatment of tilapia fillets, before they were frozen, could improve the quality of the fish during storage. These pretreatments included ultrahigh pressure treatment (Zhao et al., 2017) and the use of chemical water‐retaining agents or biological preservatives. In addition, the postfrozen quality of tilapia fillets could be improved by impregnating a phosphate water‐retaining agent before frozen storage (Li et al., 2020). However, recent studies have shown that excessive phosphate intake can lead to an imbalance in calcium and phosphorus (Guo et al., 2020); thus, a new type of phosphate‐free water‐retaining agent and its optimal formulation are required.

At present, many studies have found that trehalose (Gao et al., 2020), potassium bicarbonate (KHCO_3_), and chitosan (Zhang et al., 2020) have water retention properties. Trehalose molecules contain a large number of hydroxyl groups that can bind to proteins, preventing their aggregation and denaturation. Simultaneously, the free hydroxyl groups in carbohydrates such as trehalose can also interact with water, reducing the eutectic point of the system, and protecting the proteins (Pan et al., 2009). Tee and Siow (2014) treated Spanish mackerels with trehalose and a commercial combination antifreeze. The results showed that the water‐holding capacity of the treated mackerels was significantly higher than that of the control group, and the loss rate of thawed water from the treated mackerels was lower than from the control group.

In muscle tissue, pH changes can cause electrostatic repulsion between peptide chains and myofibrils. This leads to swelling of the protein structure and the ability to hold more water, thus improving water retention. Furthermore, a higher pH leads to an increase in the negative charge on proteins and thus, an increase in water molecule binding (Andersen & Jogensen, 2004). Sodium bicarbonate functions as an acidity regulator in muscle tissue by adjusting the pH (Chantarasuwan et al., 2011). Chantarasuwan et al. (2011) found that mixtures of 2.5% NaCl and 2.0% NaHCO_3_ could increase the activity of Ca^2+^‐ATPase and Mg^2+^‐ATPase in *Penaeus vannamei* (whiteleg shrimp now named *Litopenaeus vannamei*). Magnus ÅSli et al. (2012) found that the water‐holding capacity and quality of cod fillets could be improved by injecting mixtures of NaCl and NaHCO_3_ into the fillets.

Chitosan is an animal‐derived antistaling agent, which can inhibit the growth of microorganisms. Chitosan can form a membrane on the surface of materials and thereby hinder the transport of nutrients for microorganisms. In addition, chitosan carbon chains contain a positively charged amine group that can combine with negatively charged molecules, such as proteins (Dutta et al., 2009), resulting in an increase in the space between myofibrils and reducing the loss of water‐soluble proteins (Chantarasataporn et al., 2013). Therefore, chitosan can improve water retention and the texture of tissue (Hajidoun & Jafarpour, 2013). Soares et al. (2013) found that 0.5% and 0.75% chitosan not only reduced weight loss in salmon but also effectively inhibited lipid oxidation. There are no reports describing the use of mixtures of trehalose, KHCO_3_, and chitosan and, in particular, whether the three together can enhance the nutritional value of frozen tilapia fillets.

During the thawing of frozen food products, internal ice crystals melt but this water is rarely fully absorbed by the original tissue cells (Sherif, 2011). The percentage of water lost to total fillet mass is the thawing loss rate, also known as the dripping loss. The juices flowing out of the tissue contain water, proteins, inorganic salts, and water‐soluble vitamins. Thus, the thawing loss rate can measure changes in the nutritional quality of frozen foods. Rheology and structural properties (geometry and surface) of foods reflect the physical properties of the food itself, perceived by force, touch, vision, and hearing. Besides the nutritional value of food, texture characteristics are also key factors that influence consumer choices.

In this study, we investigated the effects of various concentrations of trehalose, KHCO_3_, and chitosan on the water‐holding capacity of tilapia fillets to select the appropriate concentration of water‐holding agent to maximize quality during frozen storage. Low‐field nuclear magnetic resonance (LF‐NMR; Bertram & Andersen, 2004) was used to detect muscle water‐holding capacity (María et al., 2011; Mortensen et al., 2006), and an orthogonal experimental method was used to optimize the formula and ratio for the water‐retaining agent for tilapia fillets. Our findings should provide some reference for the storage of similar aquatic products.

## 2 MATERIALS AND METHODS

### 2.1 Experimental materials and reagents

Fresh tilapia weighing 750 ± 50 g were purchased from a market in the Xia shan District (Zhanjiang City, Guangdong Province, China). The fillets were wrapped in ice and quickly transported to the laboratory; the entire process took no more than 30 min.

According to previous authors (Sánchez‐Valencia et al., 2015) and pre‐experiments in our laboratory, the suitable concentration of trehalose for storage is 1%–8% (m/v). Therefore, the dosage range of each component of the water‐retaining agent we used was as follows: trehalose 2%–8% (m/v), KHCO_3_ 0.5%–2.5% (m/v), and chitosan 0.5%–2.5% (m/v). Food‐grade trehalose and KHCO_3_ were purchased from the Food Ingredients Centre. Water‐soluble chitosan (deacetylation ≥ 85%) was purchased from Biotechnology Co., Ltd. An ATP enzyme kit was purchased from the Nanjing Institute of Bioengineering. All of these reagents were analytically pure.

### 2.2 Instruments and equipment

A UV‐Vis spectrophotometer (UV‐8000A, Shanghai Yuan Analytical Instrument Co., Ltd.), NMR cross‐linking densitometer (MicroMR, Shanghai Neumai Electronic Technology Co., Ltd.), texture analyzer (TMS‐PRO, American FTC Corporation), pH meter (PHS‐3C, Shanghai Instrument and Electrical Science Instrument Co., Ltd.), high‐speed freezing centrifuge (GTR22‐1, Beijing Times Beili Centrifuge Co., Ltd.), multichannel temperature tester (JK‐24U, Changzhou Jinai Lian Electronic Technology Co., Ltd.), homogenizer (Type 125, Shanghai Yiken Machinery Equipment Co., Ltd.), constant‐temperature water bath (HHS, Shanghai Bo Xun Industrial Co., Ltd.), suction filter (EYEL4A‐1000S, Shanghai Ailang Instrument Co., Ltd.), and an analytical balance (AUY220, Japan Shimadzu Instruments Co., Ltd.) were used in this study.

### 2.3 Material pretreatment

Healthy and fresh tilapia were butchered, peeled, and sliced. The tilapia fillets were trimmed to a length of 100 ± 5 mm, width of 60 ± 5 mm, thickness of 10 ± 2 mm, and weighed about 70 g (Zhi et al., 2019).

Trehalose solutions (2%, 5%, and 8%), KHCO_3_ solutions (1%, 1.5%, 2.0%, 2.5%, and 3.0%), and chitosan solutions (0.5%, 1%, 1.5%, 2.0%, and 2.5%) were prepared in advance and stored at 4°C until use. Each fish fillet was impregnated with 100 ml of solution, ensuring that the solution fully covered the fish fillet and incubated for 1 h at 4°C. The pretreated tilapia fillets were packed separately in polyethylene bags, sealed, and the fillets stored in a −20 ± 1°C freezer for 3 months.

### 2.4 Single‐factor test

The indices of weight gain rate, thawing loss rate, pH, and dry consumption were measured for the tilapia fillets before and after storage at −20°C for 3 months (Song et al., 2016). The effects of various concentrations of trehalose, KHCO_3_, and chitosan on the quality of tilapia fillets were investigated, and the most suitable concentration range was determined for each component.

### 2.5 Orthogonal test design

Following the results of the single‐factor test, an L_8_(2^7^) orthogonal experimental approach was used to examine the interaction among trehalose, KHCO_3_, and chitosan. The optimal combination of a phosphorus‐free water‐retaining agent formula was obtained by the L_8_(2^7^) orthogonal experiment.

### 2.6 Index determination method

#### 2.6.1 Determining the weight gain rate of soaking (coating)

Following the modified method of Zeng (2012), tilapia fillets were dried with a kitchen paper towel, weighed, and then divided into several groups to perform parallel experiments. The tilapia fillets were placed in 100 ml trehalose or KHCO_3_ solution, at a prepared concentration, placed at 4°C for 1 h, and stirred slowly every 15 min. Alternatively, the fillets were impregnated with 100 ml of a chitosan solution (at a prepared concentration) and incubated for 10 min at 4°C. The solution was drained through a gauze net and the fillets were weighed. The weight gain rate of the tilapia fillet after soaking (coating) was calculated using Equation (1).

(1) $$W\left(\%\right)=\frac{m_{1}‐m_{2}}{m_{2}}\times 100$$

where *W* is the weight gain rate (%), *m*_1_ is the mass of fish pieces after soaking (coating), and *m*_2_ is the mass of fish pieces before soaking (coating).

#### 2.6.2 Determination of water‐holding capacity

Following the modified method of Lakshmanan et al. (2007), 2.00 ± 0.01 g of minced fish meat was crushed using a homogenizer for 1 min at 4°C, placed in a preweighed centrifuge tube lined with two layers of preweighed filter paper (25 μm pore size and 0.2 mm thick), and centrifuged for 10 min at 149.85 *g* at 10°C. The mass of the filter paper was then measured after centrifugation. The water‐holding capacity was calculated using Equation (2).

(2) $$WHC\left(g/100g\right)=\left(1‐\frac{m_{2}‐m_{1}}{m}\right)\times 100$$

where *m*_2_ is the mass of filter paper after centrifugation (g), *m*_1_ is the mass of filter paper before centrifugation (g), and *m* is the mass of fish meat (2.00 ± 0.01 g).

#### 2.6.3 Determination of thawing loss rate

Following the American Association of Official Analytical Chemists (AOAC) methods (Dossou et al., 2014), thawing loss rate was calculated using Equation (3).

(3) $$W\left(\%\right)=\frac{m_{1}‐m_{2}}{m_{1}}\times 100$$

where *W* is the loss rate of thawing (%), *m*_1_ is the mass of fish before freezing (g), and *m*_2_ is the mass of fish after thawing (g).

#### 2.6.4 Determination of pH values

Minced fish meat (10 g) was placed in a beaker and 100 ml of distilled water was added. The mixture was homogenized for 1 min, left to rest for 30 min, and filtered using the same procedure as described in Section 2.6.2. The pH was determined using a pH meter (Xia, 1993).

#### 2.6.5 Determination of Ca^2+^‐ATPase activity

Following the instructions of the ATP enzyme test information provided by the manufacturer and developed by Nanjing Bioengineering Institute, Ca^2+^‐ATPase activity was measured and expressed as the micromoles of inorganic phosphorus produced per milligram protein per hour (μmol Pi/mg prot·h) at 25°C.

#### 2.6.6 Determination of texture (“chewiness”)

A TMS‐Pro texture analyzer (Wang et al., 2011) using the TPA model (Texture Analyser carries its own mode) was used to simulate human teeth chewing food to evaluate the texture characteristics (chewability) of the pretreatment fish fillets. The specific experimental conditions included using a flat‐bottom cylindrical probe P/5 (5 mm diameter), test speed of 60 mm/min, compression degree 50%, dwell time 5 s, and ambient temperature 18–20°C.

#### 2.6.7 Determination of water relaxation time and relaxation intensity

The frozen tilapia slices were thawed in 4°C refrigerators for 24 h. Tissue pieces (2.5 g mass and 1 × 1 × 1 cm volume) were prepared and placed into the NMR tube (17.0 mm diameter) for LF‐NMR determination. The following parameters were set: main frequency 22 MHz, RF signal frequency offset O1 = 840,899.4 Hz, sampling points TD = 1024, sampling frequency SW = 200 kHz, sampling starting point D3 = 80 μs, time interval TR = 1000 ms, repeated sampling times NS = 4, half echo time τ = 100 μs, and echo number Echo Cnt = 1800. After detection, the distribution of the transverse relaxation time *T*_2_ was inverted. The Carr–Purcell–Meiboom–Gill (CPMG) sequence was used to detect the transverse relaxation process of the samples. The mathematical expression of the relaxation signal is shown in Equation (4).

(4) $$M\left(t\right)=\sum_{i}p_{i}exp\left(‐\frac{t}{T_{2i}}\right)$$

where *M*(t) is the signal quantity after the transverse magnetization vector decays to time *t* and *p_i_* is the signal strength of the i^th^ component in the sample. The total signal size is the sum of the signal sizes generated by all components. *T*_2i_ represents the lateral relaxation time of the i^th^ component in the sample.

#### 2.6.8 Data processing and statistical analysis

All test results were averaged from triplicate values and expressed as the mean ±standard deviation (SD). The Origin 8 (Origin Lab Corporation) software was used to map the data and JMP 7 software was used for univariate analysis of variance along with a Duncan multiple comparison test (*p* < .05 was significant).

## 3 RESULTS AND DISCUSSION

### 3.1 Effect of trehalose concentration on water retention of tilapia fillets

**FIGURE 1:**
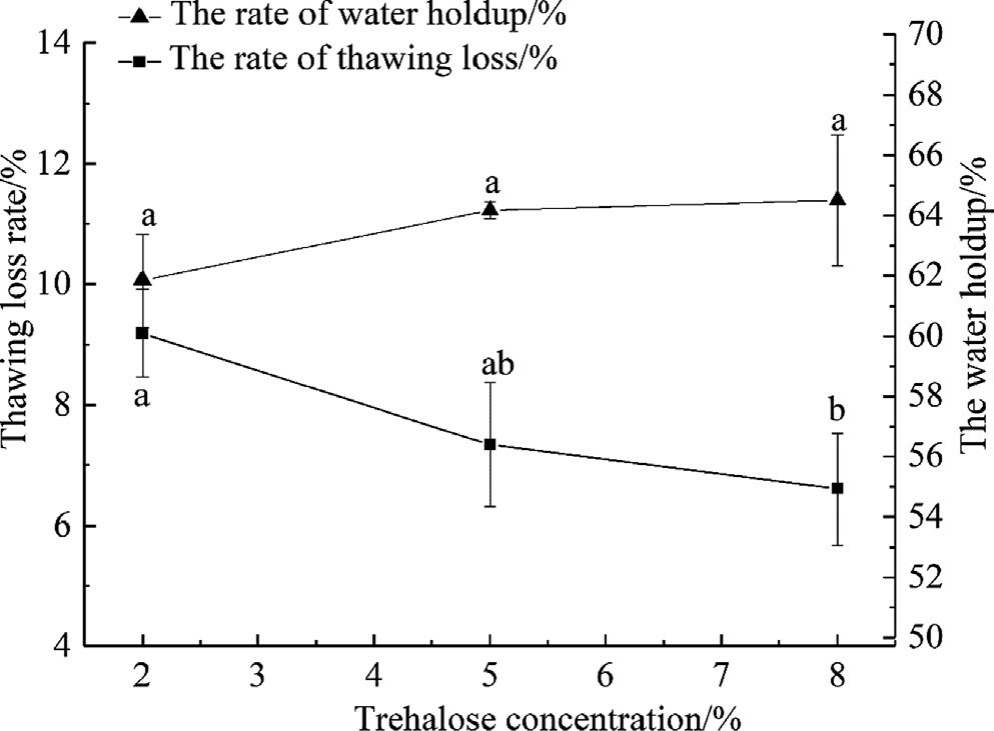
Effects of different concentrations of trehalose on the thawing loss and WHC of tilapia fillets

The thawing loss rate is an important index to measure the water retention performance of fish. Thawing loss is mainly due to growth of ice crystals inside the fish during the freezing process, which puncture the cell membranes leading to dehydration and aggregation of protein. This results in the denaturation of protein and the loss of water diversion (Du et al., 2016). Figure 1 shows the changes in thawing loss rate and water‐holding capacity of the tilapia fillets treated with different concentrations of trehalose after 3 months of frozen storage. An increase in trehalose concentration resulted in a significant decrease in thawing loss rate from 9.18% to 6.60% (*p* < .05). The water‐holding capacity increased gradually, but there was no statistical difference between treatments, which might be due to the similarity of trehalose concentrations. In addition, trehalose concentrations of 5% and 8% could reduce the thawing loss rate and maintain more water in the tilapia fillets compared to trehalose concentrations of 2%; however, there was no significant difference between the two trehalose concentrations (*p* > .05). These results were similar to those reported by Ma et al. (2015). Therefore, it can be seen that 5%–8% trehalose can be used to maintain the internal moisture content of tilapia fillets. At the same time, Zhou et al. (2006) also found that the tilapia surimi is treated with 8% trehalose, which can reduce the protein denaturation of frozen surimi and improve the water retention rate of surimi. In addition, Nopianti et al. (2012) also found that trehalose can improve the freezing resistance of threadfin bream surimi. It can be concluded that the quality of frozen aquatic products can be improved after aquatic products are treated with appropriate concentration of trehalose.

### 3.2 Effect of KHCO_3_ concentration on water retention of tilapia fillets

**FIGURE 2:**
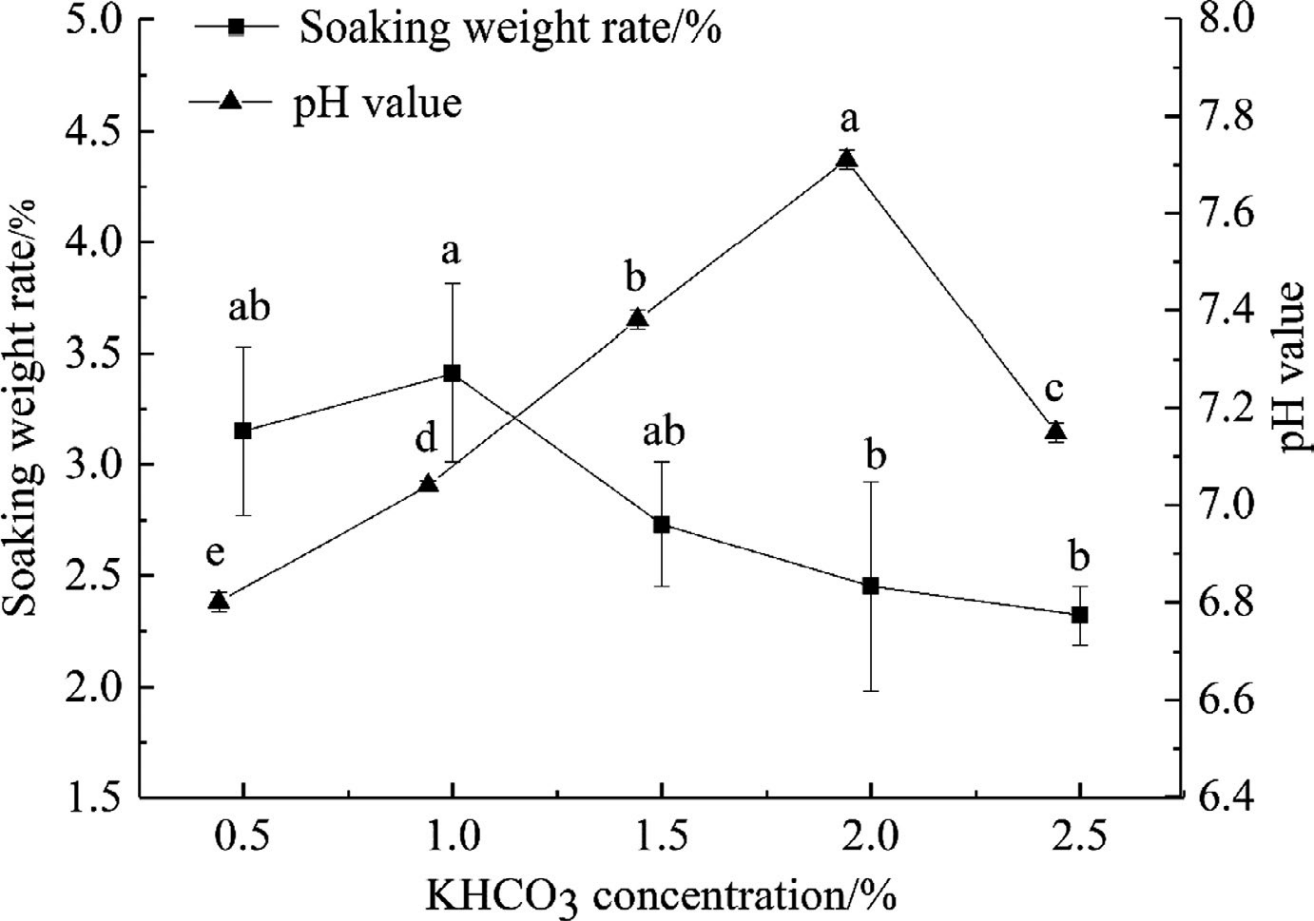
Effects of different concentrations of KHCO_3_ on the weight gain and pH value of tilapia fillets

The soaking weight gain rate and pH values of tilapia fillets after treatment with different concentrations of KHCO_3_ are shown in Figure 2. The change in soaking weight gain rate and pH value were significantly affected by KHCO_3_ concentration (*p* < .05). Here, increasing the concentration of KHCO_3_ initially resulted in an increased rate of fish weight gain, followed by a decrease. More specifically, 1.0% KHCO_3_ resulted in a 3.41% increase in weight of the fish fillets, but then it gradually decreased. The high KHCO_3_ concentration difference inside and outside of the fish decreased, most likely due to the greater osmotic pressure outside of the cell than inside the cell (Gao et al., 2009). Thus, the cells lost water, resulting in a decrease in weight gain rate. However, the weight of the fish pieces after immersion still increased. The pH value increased with the increase in KHCO_3_ concentration, and the water‐holding capacity of fillets increased gradually. When the concentration of KHCO_3_ was 2.0%, the maximum pH was 7.71, and the effect was significant (*p* < .05).

Therefore, concentrations of KHCO_3_ between 1.0% and 1.5% are suitable for water conservation and weight gain in tilapia fillets. However, when the concentration is >1.0%, the cooked product will have an bitter taste, which has a damaging effect on the flavor of the product (Zhang et al., 2018). Thus, the concentration of KHCO_3_ should be 1.0%.

### 3.3 Effect of chitosan concentration on water retention of tilapia fillets

**FIGURE 3:**
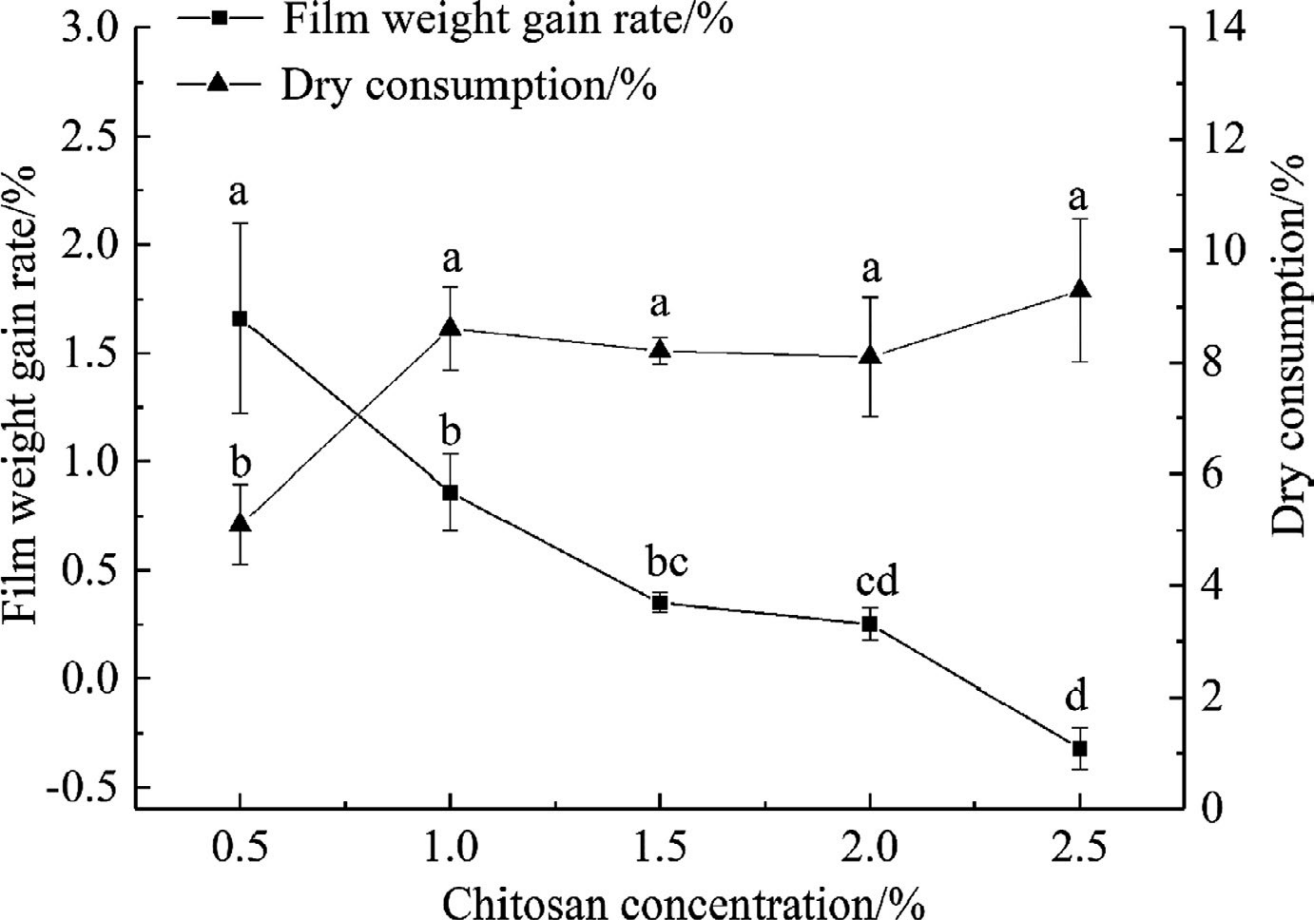
Effects of different concentrations of chitosan on the weight gain and loss of tilapia fillets

The change in film weight gain rate of tilapia fillets after chitosan treatment and the dry consumption rate of fillets after frozen storage for 3 months are shown in Figure 3. An increase in chitosan concentration causes the weight gain of fish fillets to decrease gradually. The weight gain rate using 0.5% chitosan was 1.66%, which was significantly higher than that of the other experimental groups (*p* < .05), whereas the weight gain rate of tilapia fillets coated with 2.5% chitosan was −0.32%. This can be explained by the higher external chitosan concentration drawing water from inside the fish fillets outwards (Gao et al., 2009). Chitosan, being a large macromolecule, cannot enter the fish fillet; this results in a reduction in fish fillet mass.

Furthermore, ice crystals on the fish surface will sublimate and dry consumption will occur during freezing (Chen et al., 2014). After 3 months of storage at −20°C, the quality of the tilapia fillet decreased, and the dry consumption was 5.10%–9.29%. The dry consumption of the fish fillet coated with 0.5% chitosan was 5.10%, significantly lower than that of the other experimental groups (*p* < .05), which was consistent with the largest coating weight gain rate result. Therefore, in this experiment, 0.5% chitosan could prevent the dry consumption of the fish fillets to a great extent, while the coating effect was the best.

### 3.4 Effects of different water‐retaining agents on the moisture state of frozen tilapia fillets

**FIGURE 4:**
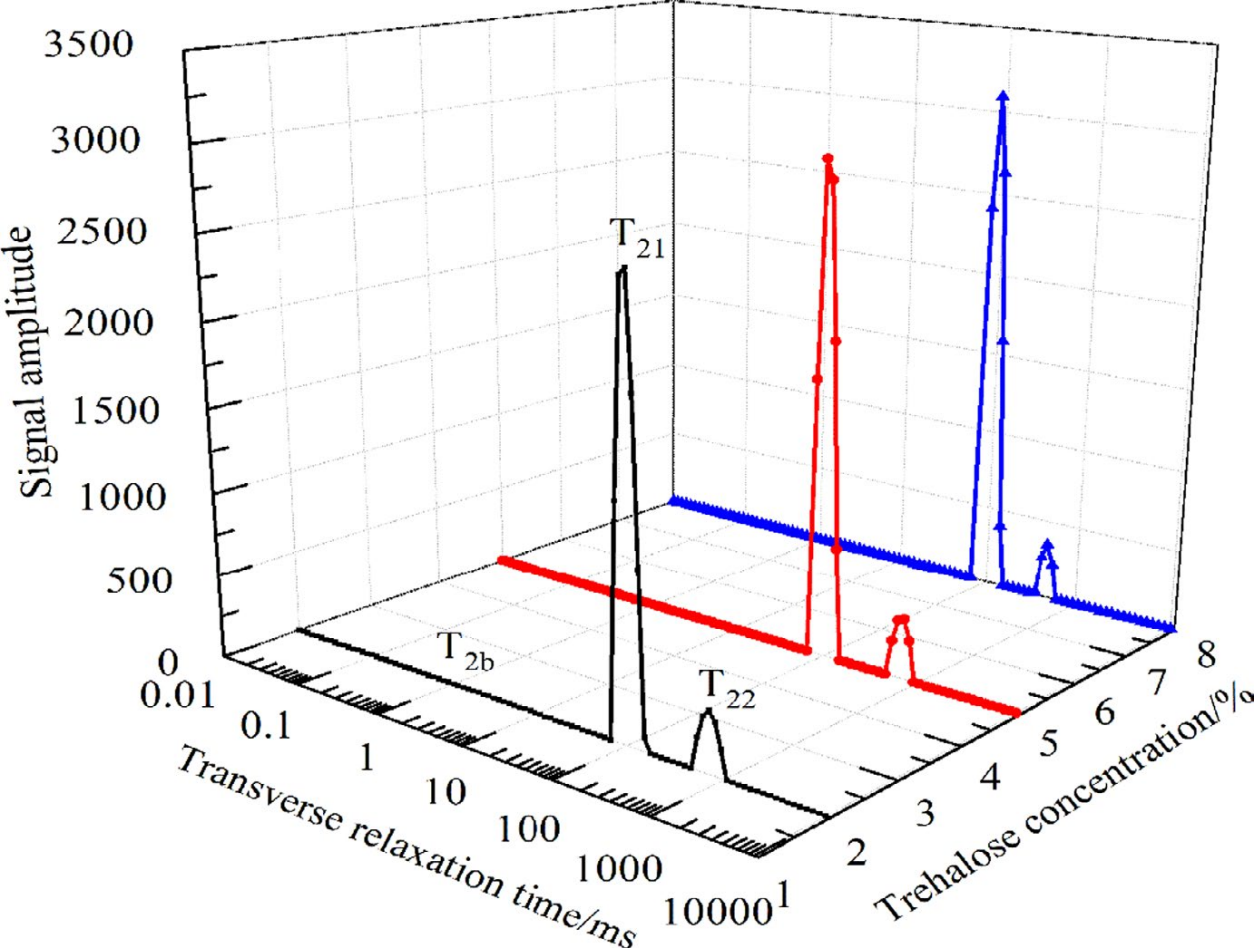
Effects of trehalose on transversal relaxation time *T*_2_ of tilapia fillet

When a proton is in a different chemical environment, the transverse relaxation time *T*_2_ varies. The smaller the *T*_2_ value, the smaller the degree of freedom the proton has; thus, *T*_2_ can reflect the fluidity of water molecules (Bertram & Andersen, 2007). Figure 4 shows the water state of tilapia fillets treated with trehalose, as detected using LF‐NMR. Three peaks are shown in the lateral relaxation time during frozen storage, which indicate the three forms of water in the tilapia muscle. These three forms represent: water closely bound with macromolecules, that is, binding water *T*_2b_ (0.1–1.0 ms); water inside the myofibril, that is, water that cannot easily flow *T*_21_ (30–200 ms); and water outside the myofibril network, that is, free water *T*_22_ (300–800 ms) (Bertram et al., 2002). The area of each peak, enclosed by the abscissa, is the water content of each part (Luo et al., 2021). Thus, the corresponding water content can be obtained by integrating each peak. The combined water exhibited no change during the frozen storage process, and its percentage was low. Here, Table 1 shows the combined water and *T*_21_, combined to calculate the percentage content of water and free water in the fish stored for 3 months.

**TABLE 1:** Effects of additives on relative percentage of different kinds of water in tilapia fillets

Treatment conditions	A_21_%	A_22_%
2% trehalose	48.36 ± 3.97^b^	51.64 ± 3.97^a^
5% trehalose	69.95 ± 5.74^a^	30.05 ± 5.74^b^
8% trehalose	67.22 ± 3.64^a^	32.78 ± 3.64^b^
0.5% KHCO_3_	93.81 ± 2.29^a^	6.19 ± 2.29^b^
1.0% KHCO_3_	88.68 ± 0.33^ab^	11.32 ± 0.33^ab^
1.5% KHCO_3_	84.54 ± 4.70^b^	15.46 ± 4.70^a^
2.0% KHCO_3_	85.06 ± 1.69^b^	14.94 ± 1.69^a^
2.5% KHCO_3_	85.97 ± 2.33^b^	14.03 ± 2.33^a^
0.5% chitosan	91.43 ± 0.42^a^	8.57 ± 0.42^a^
1.0% chitosan	91.93 ± 1.56^a^	8.07 ± 1.56^a^
1.5% chitosan	90.12 ± 1.43^a^	9.88 ± 1.43^a^
2.0% chitosan	93.02 ± 0.55^a^	6.98 ± 0.55^a^
2.5% chitosan	90.38 ± 1.37^a^	9.62 ± 1.37^a^

Note

a Indicates significant difference.

Table 1 shows that the content of water A_21_ in 5% and 8% trehalose‐treated fish meat was larger than that in 2% trehalose‐treated fish (*p* < .05), and the content of free water A_22_ was smaller than that in 2% trehalose‐treated fish (*p* < .05). Thus, 5%–8% trehalose can cause myofibrillar protein to bind more water and improve water‐holding capacity, which is consistent with the previous conclusion. In addition, the results are consistent with Huang's (2020) results that trehalose can reduce the degeneration of myofibrin in frozen silver carp. With the increase in KHCO_3_ concentration, A_21_ decreased from 93.81% to 85.97% (*p* < .05), while A_22_ increased from 6.19% to 14.03% (*p* < .05). Thus, the water in the protein reticular structure gradually migrated outwards (Hao et al., 2009). However, there was no significant difference between 0.5% and 1.0% KHCO_3_ treatment (*p* > .05). The water content of A_21_ was higher; therefore, 0.5%–1.0% KHCO_3_ was the best choice. The A_21_ and A_22_ were not significantly affected by different concentrations of chitosan (*p* > .05). However, the water content of fish protein was higher after the chitosan coating. Therefore, combined with the previous experimental results and considering the experimental cost, 0.5% chitosan is the best choice.

### 3.5 Orthogonal experimental results and analysis of the water‐retaining agent

According to the single‐factor test of trehalose, KHCO_3_, and chitosan, and the NMR experiment, our studies found that tilapia fillets pretreated with appropriate concentration of trehalose, KHCO_3_, and chitosan before storage can better retain the internal water of the tilapia fillet. But the single‐factor approach simply considered that the effect of the three different concentrations of water‐retaining agent on the internal moisture of tilapia fillets during frozen storage, and it did not consider the effect on the proteins. However, protein is the key factor affecting the quality of tilapia, so it is necessary to study the changes in proteins in tilapia fillets during frozen storage. In this study, the activity of Ca^2+^‐ATPase was measured to characterize changes in proteins since Ca^2+^‐ATPase activity can represent changes in myosin head properties and it can also be widely used to evaluate myosin integrity (Hu et al., 2014).

According to the single‐factor test of trehalose, KHCO_3_, and chitosan, the optimal concentration is 5%–8%, 1.0%, and 0.5%, respectively. The factor levels of composite additives are shown in Table 2. Considering the interactions among trehalose, KHCO_3_, and chitosan, the orthogonal test table of L_8_(2^7^) was used and arranged according to the interaction list. The interaction between A, trehalose, and B, KHCO_3_, is A × B, while the other two groups are A × B and A × C, with C being chitosan. The results are shown in Table 3.

**TABLE 2:** The factor and level of Taguchi experiment of mixed cryoprotectants

Level	Factors		
	A Trehalose/%	B KHCO/%	C Chitosan/%
1	4	0.8	0.2
2	8	1.2	0.8

**TABLE 3:** Range analysis result of Taguchi experiment of mixed cryoprotectants

Test No.	A	B	A × B	C	A × C	B × C	Empty column	Ca^2+^‐ATPase activity /μmolPi/mgprot/h	Chewiness/mJ	Soaking weight gain rate/%
1	1	1	1	1	1	1	1	1.84	6.55	3.65
2	1	1	1	2	2	2	2	1.68	8.32	3.94
3	1	2	2	1	1	2	2	1.53	3.78	4.13
4	1	2	2	2	2	1	1	1.32	3.84	4.58
5	2	1	2	1	2	1	2	1.33	6.09	2.80
6	2	1	2	2	1	2	1	1.42	7.06	3.09
7	2	2	1	1	2	2	1	1.38	8.85	1.97
8	2	2	1	2	1	1	2	1.38	7.69	2.21
K_1j_	6.372	6.264	6.271	6.081	6.174	5.874	5.960			
K_2j_	5.510	5.617	5.610	5.801	5.707	6.007	5.922			
k_1j_	1.593	1.566	1.568	1.520	1.544	1.469	1.490			
k_2j_	1.377	1.404	1.403	1.450	1.427	1.502	1.481			
R_j_	0.215	0.162	0.165	0.070	0.117	0.033	0.009			
Primary and secondary order Ca^2+^‐ATPase activity A > A × B > B > A × C > C > B × C										
K_1j_	22.488	28.028	31.410	25.270	25.088	24.173	26.295			
K_2j_	29.693	24.153	20.770	26.910	27.093	28.008	25.885			
k_1j_	5.622	7.007	7.853	6.318	6.272	6.043	6.574			
k_2j_	7.423	6.038	5.193	6.728	6.773	7.002	6.471			
R_j_	1.801	0.969	2.660	0.410	0.501	0.959	0.103			
Primary and secondary order Chewiness A × B > A > B > B × C > A × C > C										
K_1j_	16.313	13.482	11.779	12.549	13.089	13.244	13.287			
K_2j_	10.061	12.893	14.595	13.826	13.285	13.130	13.088			
k_1j_	4.078	3.370	2.945	3.137	3.272	3.311	3.322			
k_2j_	2.515	3.223	3.649	3.456	3.321	3.282	3.272			
R_j_	1.563	0.147	0.704	0.319	0.049	0.029	0.050			
Primary and secondary order Soaking weight gain rate A > A × B > C > B > A × C > B × C										

Ca^2+^‐ATPase activity, chewiness, and soaking weight gain of tilapia fillets were used as indices to evaluate the effects of different additives on the quality of tilapia fillets. Myofibrillar protein Ca^2+^‐ATPase activity is an important index to evaluate the integrity of myofibrillar protein. The higher the Ca^2+^‐ATPase activity, the better the integrity of myofibrillar protein and the lower the degree of protein denaturation.

The texture profile method is used to analyze the texture of fish meat, with texture characteristics of the product described by classification. Hardness is the magnitude of the force needed to bite into food, elasticity is the ability of food to return to its original shape after deformation, cohesion represents the strength of the internal bonds that make up the sample and reflects the integrity of the food when chewed, and chewiness refers to the amount of work done when the sample is chewed before swallowing. Chewiness is numerically equal to the product of hardness, elasticity, and cohesion, whereas the weight gain rate of immersion reflects the increase in fish quality after immersion (Guan & Li, 2010).

Table 3 shows the orthogonal test results of the composite additives and their range analysis results, with Ca^2+^‐ATPase activity as the index. According to the range Rj, the antifreeze protection effect of various factors on tilapia fillet is A > A × B > B > A × C > C > B × C, and the optimal combination is A_1_B_1_C_1_. According to the analysis of variance in Table 4, the results of the *F* test show that factor A and interaction A × B are significant (*p* < .05), while factor B, factor C, factor A × C, and the interaction B × C are not significant (*p* > .05). Given that factor A is more important than the interaction A × B, priority should be given to A’s superior level, that is, A_1_.

**TABLE 4:** Analysis of variance of Taguchi experiment

Variation source	SS	*df*	*F* ratio	*p* value
Ca^2+^‐ATPase activity				
A	0.092	1	462.25	.0296^a^
B	0.054	1	272.25	.0385^a^
A × B	0.058	1	289.00	.0374^a^
C	0.010	1	49.00	.0903
A × C	0.026	1	132.25	.0552
B × C	0.002	1	12.25	.1772
Empty column	0.0002	1		
Total	0.24	7		
Chewiness				
A	6.480	1	293.878	.0371^a^
B	1.862	1	84.465	.0690
A × B	14.151	1	641.778	.0251^a^
C	0.336	1	15.247	.1596
A × C	0.510	1	132.25	.1305
B × C	1.843	1	23.132	.0694
Empty column	0.022	1		
Total	25.183	7		
Soaking weight gain rate				
A	4.852	1	880.111	.0215^a^
B	0.044	1	7.893	.2177
A × B	1.001	1	181.608	.0472^a^
C	0.202	1	36.574	.1043
A × C	0.006	1	1.000	.5000
B × C	0.002	1	0.274	.6928
Empty column	0.006	1		
Total	6.11	7		

Note

a Indicates significant difference.

Indicates A Trehalose/%.

Considering the three indices of Ca^2+^‐ATPase activity, chewability, soaking weight gain rate, and chitosan concentration have no significant effect on the results. Thus, C_1_ can be selected considering cost reduction and convenient operation. Therefore, the optimal combination levels of additives should be A_1_B_2_C_1_, that is, 4% trehalose + 1.2% KHCO_3_ + 0.2% chitosan.

### 3.6 Experimental verification of the optimized combination of the phosphorus‐free water‐retaining agent

We conducted a test to verify whether the best concentration combination of different water‐retaining agents (4% trehalose + 1.2% KHCO_3_ + 0.2% chitosan) influenced the water‐holding capacity of tilapia fillets and whether it could improve the quality of tilapia fillets at the same time. The fillets were treated according to the processing method in Section 2.3 and then frozen for 3 months. The samples were then thawed using a vacuum thawing method at 9 kPa vacuum degree. Ca^2+^‐ATPase activity and chewiness were used as indicators. LF‐NMR was used to analyze the water content change in fish meat, while the blank control group and natural air thawing were used as control groups. The physicochemical indices of frozen tilapia fillets after different pretreatments are shown in Table 5.

**TABLE 5:** Changes in physicochemical quality of frozen tilapia fillets by different treatment

Indicators	Control	Sample
Ca^2+^‐ATPase activity (μmolPi/mgprot/h)	0.97 ± 0.12	1.39 ± 0.19^a^
Chewiness(mJ)	4.75 ± 0.80	8.55 ± 0.60^a^
A_2b_ (%)	0.25 ± 0.08	0.18 ± 0.25
A_21_ (%)	97.59 ± 0.09	98.28 ± 0.79^a^
A_22_ (%)	2.15 ± 0.15^a^	1.21 ± 0.08

a Indicates that the difference between the blank group and the experimental group is significant at *p* < .05 level.

The Ca^2+^‐ATPase activity of the tilapia fillets in the optimized experimental group was 1.39 μmol Pi/mg prot·h (Table 5), significantly higher than that of the blank control group (*p* < .05), with an increase of 43.3%. The degree of protein denaturation of the tilapia fillets after soaking in the water‐retaining agent and vacuum thawing was relatively low compared to that of the blank control group. The chewability of the optimized experimental group was 8.55 mJ, significantly higher than that of the blank group (4.75 mJ; *p* < .05), with an increase of 80%, indicating that the tissue structure of the optimized experimental group was better. In addition, the Ca^2+^‐ATPase activity and chewability of the optimized experimental group in Table 5 showed that they were lower than the corresponding values of some groups in Table 3, mainly because the tilapias used in these experiments were purchased at different seasons. Table 5 raw materials were purchased in winter, whereas Table 3 experimental fillets were purchased at the end of spring. Different fat content of tilapia in different seasons may have led to this result, but it still did not affect the conclusion of the experiment.

The moisture distribution and percent content in the two fish fillet groups were measured using LF‐NMR (Table 5). There was no difference in the percentage of combined water between the two groups (*p* > .05); that is, after 3 months of frozen storage, there was no change in the part of combined water of the fish meat. In addition, the level of A_21_ in the experimental group was significantly higher than that of the control group (*p* < .05). The results showed that the addition of 4% trehalose + 1.2% KHCO_3_ + 0.2% chitosan could lead to a higher retention of internal water content of the fish myofibrils. In addition, the appropriate defrosting method can reduce water loss and enhance the water‐holding capacity of the fish protein.

The free water ratio of the A_22_ control group was significantly higher than that of the optimized experimental group (*p* < .05), which further proved that the water‐retaining agent had a better water‐holding capacity and reduced the water migration from the inside of the fish fillet. Due to the addition of trehalose, KHCO_3_, and chitosan hydroxyl groups, there was likely an increase in the structure of the fish muscle fibrin gel network, which could increase the muscle water absorption capacity and slow down the mobility of water molecules (Sriket et al., 2007).

In this study, we found that compared with 2% trehalose, 5%–8% trehalose significantly decreased the thawing loss rate of tilapia fillets from 9.18% to 6.60% and maintained a high water‐holding capacity of about 64.00%. In addition, LF‐NMR showed that the percentage of water content in 5% and 8% trehalose‐treated fish fillets was significantly higher at 69.95% and 67.22%, respectively. Therefore, the optimal concentration range of trehalose is 5%–8%.

The weight and pH of tilapia fillets soaked in 0.5%–2.5% KHCO_3_ increased, with 1.0% KHCO_3_ showing the highest weight gain rate of 3.41%, whereas the maximum pH value of 2.0% showed 7.71, a high water‐holding capacity. Furthermore, the results of LF‐NMR showed that 0.5 and 1.0% KHCO_3_‐treated fillets had higher percentages of nonmobile water of 93.81% and 88.68%, respectively.

The weight gain rate observed following 0.5% chitosan film treatment was significantly higher than that of the other concentrations tested, reaching 1.66%. After 3 months storage, the dry consumption of the fish fillets was 5.10%, which was better than that of the experimental group. Moreover, LF‐NMR analysis showed there was no significant change in the water content after 0.5%–2.5% chitosan coating. Therefore, the optimum concentration of chitosan was 0.5%.

The optimum combination of phosphorus‐free water‐retaining agent was A_1_B_2_C_1_, equivalent with 4% trehalose + 1.2% KHCO_3_ + 0.2% chitosan. The optimized water‐retaining agent could inhibit the denaturation of protein and improve the frost resistance of tilapia fillets. Furthermore, it could improve the texture characteristics, enhance the chewing sense, increase the weight, and improve the water‐holding capacity of the fish fillets.

The Ca^2+^‐ATPase activity of the tilapia fillets treated with 4% trehalose + 1.2% KHCO_3_ + 0.2% chitosan and vacuum thawed was 1.39 μmol Pi/mg prot·h and the chewability was 8.55 mJ; an increase of 43.3% and 80.0%, respectively, compared with that of the control group.

## 4 CONCLUSIONS

In this study, we showed that the optimum phosphorus‐free water‐retaining agent combined with vacuum thawing can reduce the denaturation of protein and maintain high‐quality texture characteristics of tilapia fillets. Furthermore, LF‐NMR analysis showed that the treatment can slowdown water migration from inside the myofibrillar protein to the outside of the fillets. We found that using 4% trehalose + 1.2% KHCO_3_ + 0.2% chitosan can retain the quality of tilapia fillets during frozen storage and following vacuum thawing. Thus, this study can provide technical guidance for the long‐term storage of tilapia fillets and similar fish products.

## CONFLICT OF INTEREST

Authors declare no conflict of interest.

## AUTHOR CONTRIBUTION

**Min Li:** Data curation (equal); Formal analysis (equal); Funding acquisition (equal); Investigation (equal); Writing – original draft (equal); Writing – review and editing (equal). **Jing Luo:** Methodology (equal); Software (equal); Supervision (equal). **Ying Zhang:** Software (equal). **Ke Zhang:** Methodology (equal); Software (equal). **Zhi‐Qiang Guan:** Methodology (equal). **ming Chang Ling:** Funding acquisition (equal); Software (equal).

## ETHICAL APPROVAL

This investigation did not involve human or animal testing.

## Data Availability

Research data are not shared.
